# Supplementary material for: Metagenomes from Coastal Sediments of Kuwait: Insights into the Microbiome, Metabolic Functions and Resistome
Source: Microorganisms. 2023 Feb 20;11(2):531. doi: 10.3390/microorganisms11020531 (PMC9960530; doi:10.3390/microorganisms11020531)
Supplement: Supplementary file 1 [file microorganisms-11-00531-s001.zip › Supplementary Data.pdf]

**Supplementary Data**

## DNA Quality and Quantity

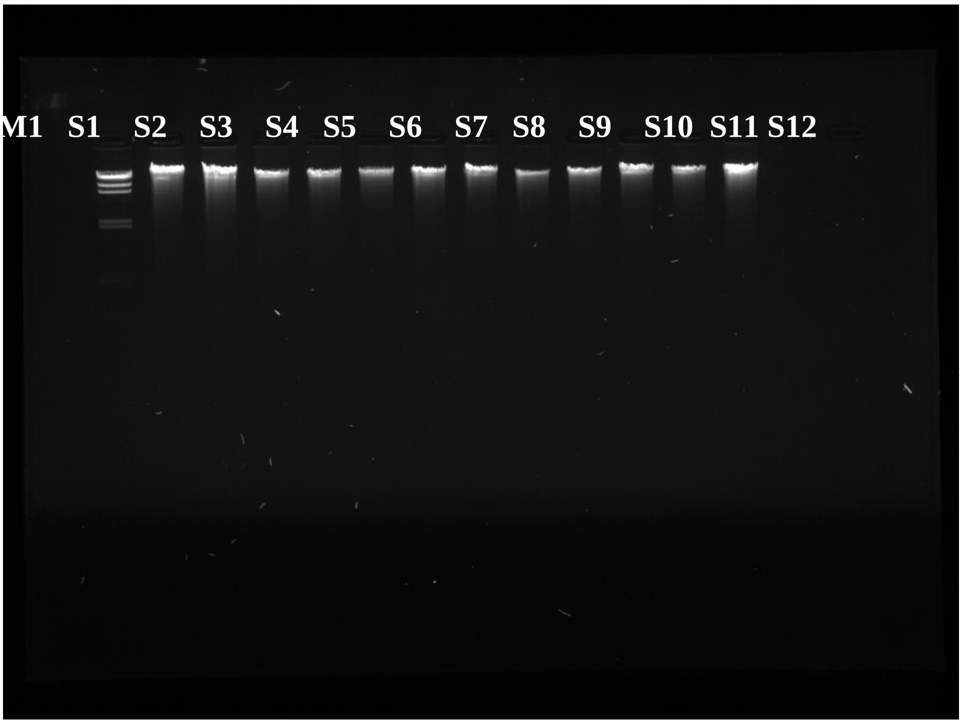


**Figure S1. Agarose gel (1.0%) showing the isolated DNA from 12 sediment samples of Kuwait, Lane M1-1Kb Marker, Lane S1-S12 represent the 12 sediment sample**

**Table S1: DNA quantity and Yield**

| # | Sample ID | Concentration ng/ul | Elution volume | Total amount (μg) |
|---|---|---|---|---|
| 1 | S1 | 31.80 | 20 | 0.636 |
| 2 | S2 | 13.12 | 20 | 0.262 |
| 3 | S3 | 11.08 | 20 | 0.221 |
| 4 | S4 | 0.64 | 20 | 0.012 |
| 5 | S5 | 12.68 | 20 | 0.253 |
| 6 | S6 | 9.04 | 20 | 0.180 |
| 7 | S7 | 49.92 | 20 | 0.998 |
| 8 | S8 | 7.96 | 20 | 0.159 |
| 9 | S9 | 18.76 | 20 | 0.375 |
| 10 | S10 | 10.24 | 20 | 0.204 |
| 11 | S11 | 15.60 | 20 | 0.312 |
| 12 | S12 | 1.55 | 20 | 0.030 |

## Raw Data and Assembly quality and statistics

The genomic DNA was randomly sheared into short fragments. The obtained fragments were end repaired, A-tailed and further ligated with Illumina adapter. The fragments with adapters were PCR amplified, size selected, and purified. The library was checked with Qubit and real-time PCR for quantification and bioanalyzer for size distribution detection. Quantified libraries were pooled and sequenced on an Illumina platform. Quality control and host filtering of the raw data were carried out to obtain effective data

**Table S2: QC and statistics of Raw sequence data**

| #Sample | RawData | RawReads(#) | Low_Q | CleanData | Clean_Q20 | Clean_Q30 | Effective(%) |
|---|---|---|---|---|---|---|---|
| S5 | 6,465.77 | 43,105,110 | 0 | 6,455.96 | 96.91 | 91.93 | 99.848 |
| S12 | 6,634.69 | 44,231,292 | 0 | 6,620.87 | 96.83 | 92.04 | 99.792 |
| S4 | 6,875.03 | 45,833,566 | 0 | 6,845.47 | 97.01 | 92.38 | 99.57 |
| S1 | 6,259.21 | 41,728,094 | 0 | 6,254.81 | 97.34 | 92.67 | 99.93 |
| S8 | 5,938.15 | 39,587,698 | 0 | 5,933.18 | 97.28 | 92.86 | 99.916 |
| S7 | 6,196.10 | 41,307,360 | 0.01 | 6,190.16 | 96.62 | 91.16 | 99.904 |
| S2 | 6,421.52 | 42,810,128 | 0 | 6,417.02 | 97.43 | 93.08 | 99.93 |
| S3 | 6,329.98 | 42,199,896 | 0 | 6,312.86 | 97.14 | 92.53 | 99.73 |
| S11 | 6,487.27 | 43,248,482 | 0 | 6,481.99 | 97.12 | 92.57 | 99.919 |
| S9 | 6,441.10 | 42,940,650 | 0 | 6,436.15 | 97.25 | 92.67 | 99.923 |
| S10 | 6,476.85 | 43,179,000 | 0 | 6,469.62 | 97.42 | 93.22 | 99.888 |
| S6 | 6,767.49 | 45,116,594 | 0 | 6,761.87 | 97.42 | 93.15 | 99.917 |

**Taxonomic Profile**

This part involved the alignment of marker gene homologs to a database of taxonomically informative gene families, using sequence or phylogenetic similarity to the database sequences (microNR database) to taxonomically annotate each metagenomic homolog (MEGAN). According to the abundance table of each taxonomic level, the Krona analysis was also performed. Figs S2a-l show the KRONA plots for each sample. More details on these figures can be viewed through this link.
file:///Users/nazimahabibi/Dropbox/My%20Mac%20(MacBook-Pro.local)/Documents/ANtibiotic%20resistance%20genes/Report-X201SC21112149-Z01-F001-B1-41/files/pictures/04.Taxonomy/taxonomy.krona.html

The KRONA plots were created using the KRONA analysis tool developed by Ondov et al., 2011 available at https://github.com/marbl/Krona/wiki/

Ondov BD, Bergman NH, and Phillippy AM. Interactive metagenomic visualization in a Web browser. BMC Bioinformatics. 2011 Sep 30; 12(1):385.

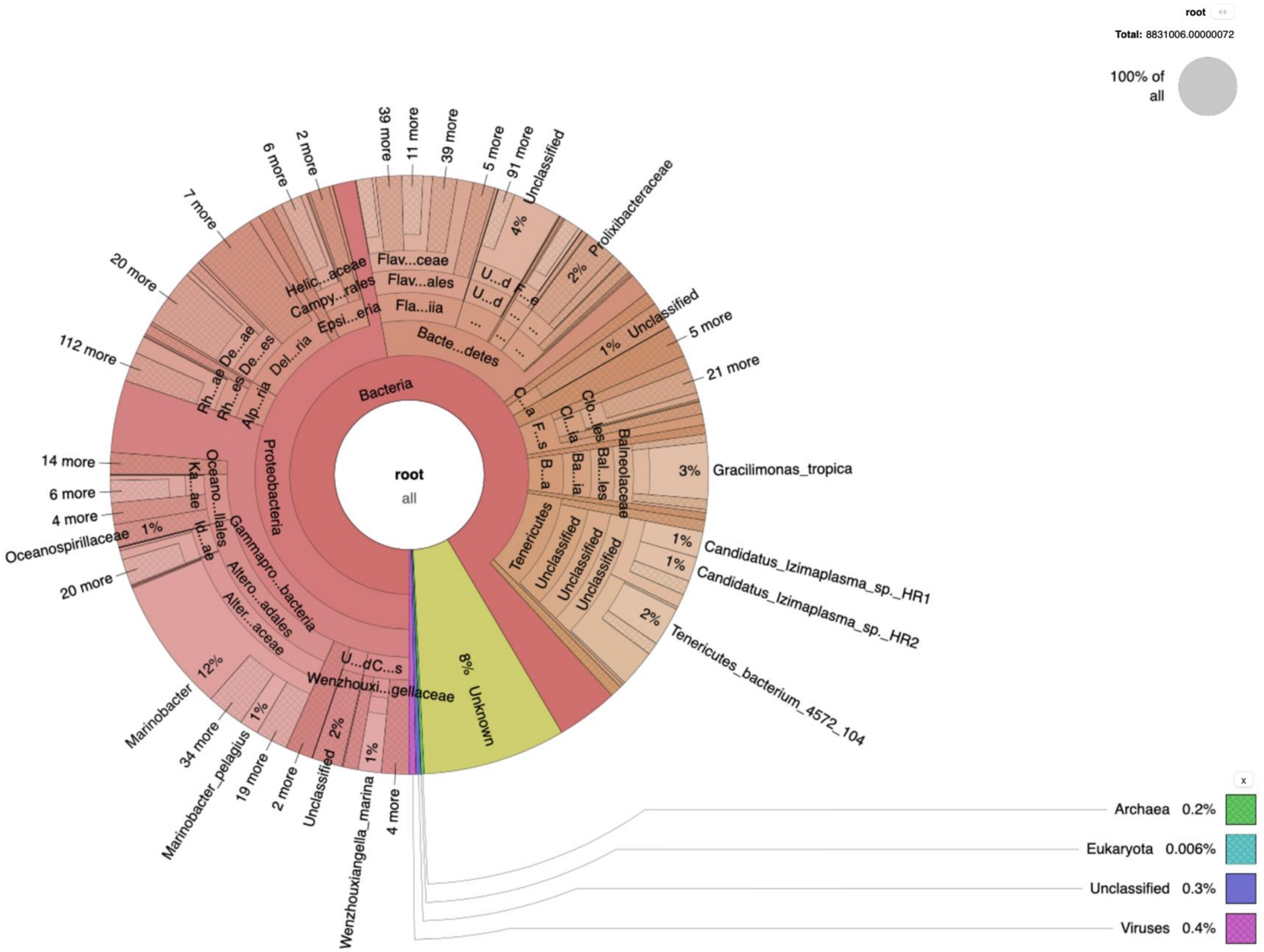


**Figure S2a. KRONA plot of sample S1**

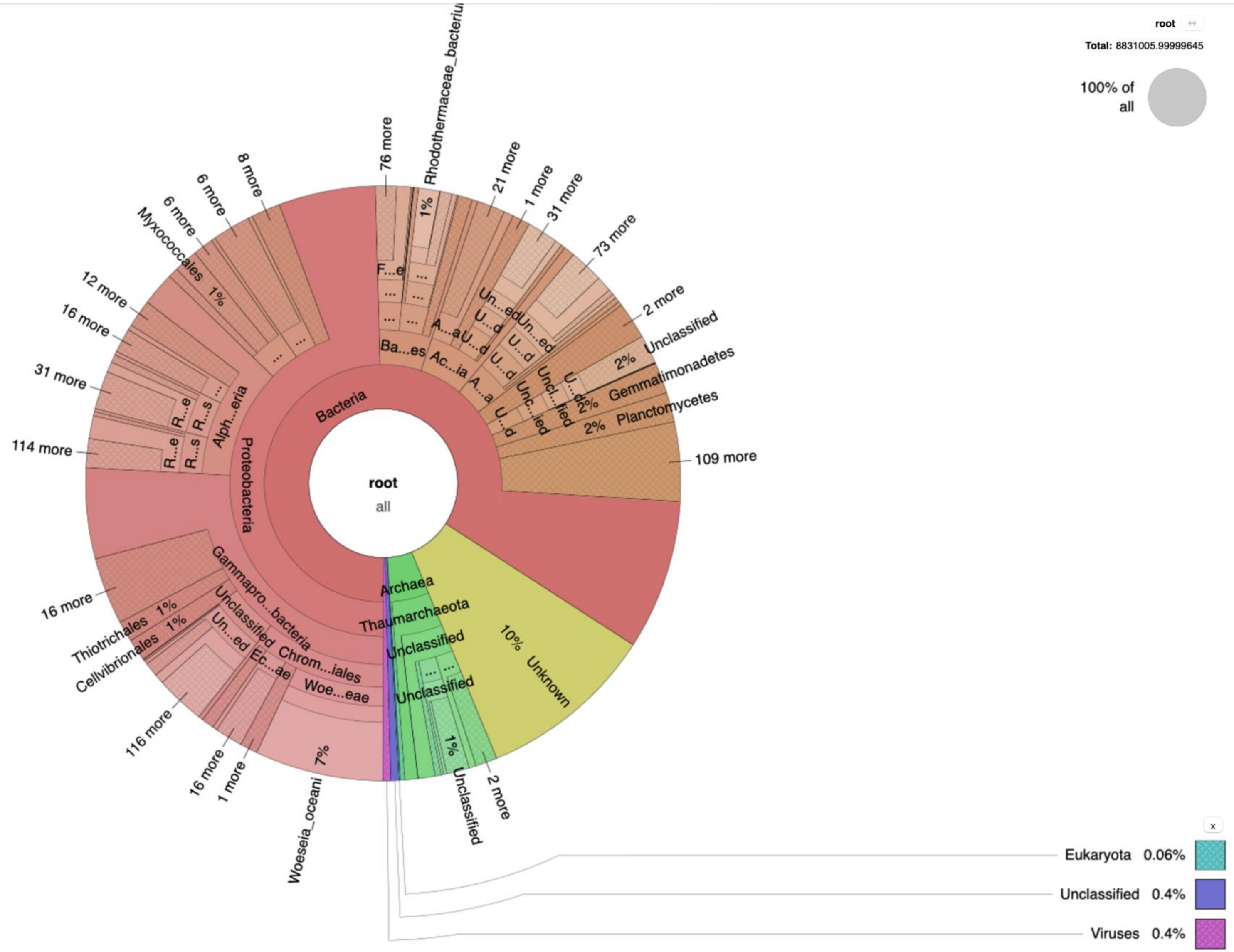


**Figure S2b. KRONA plot of sample S2**

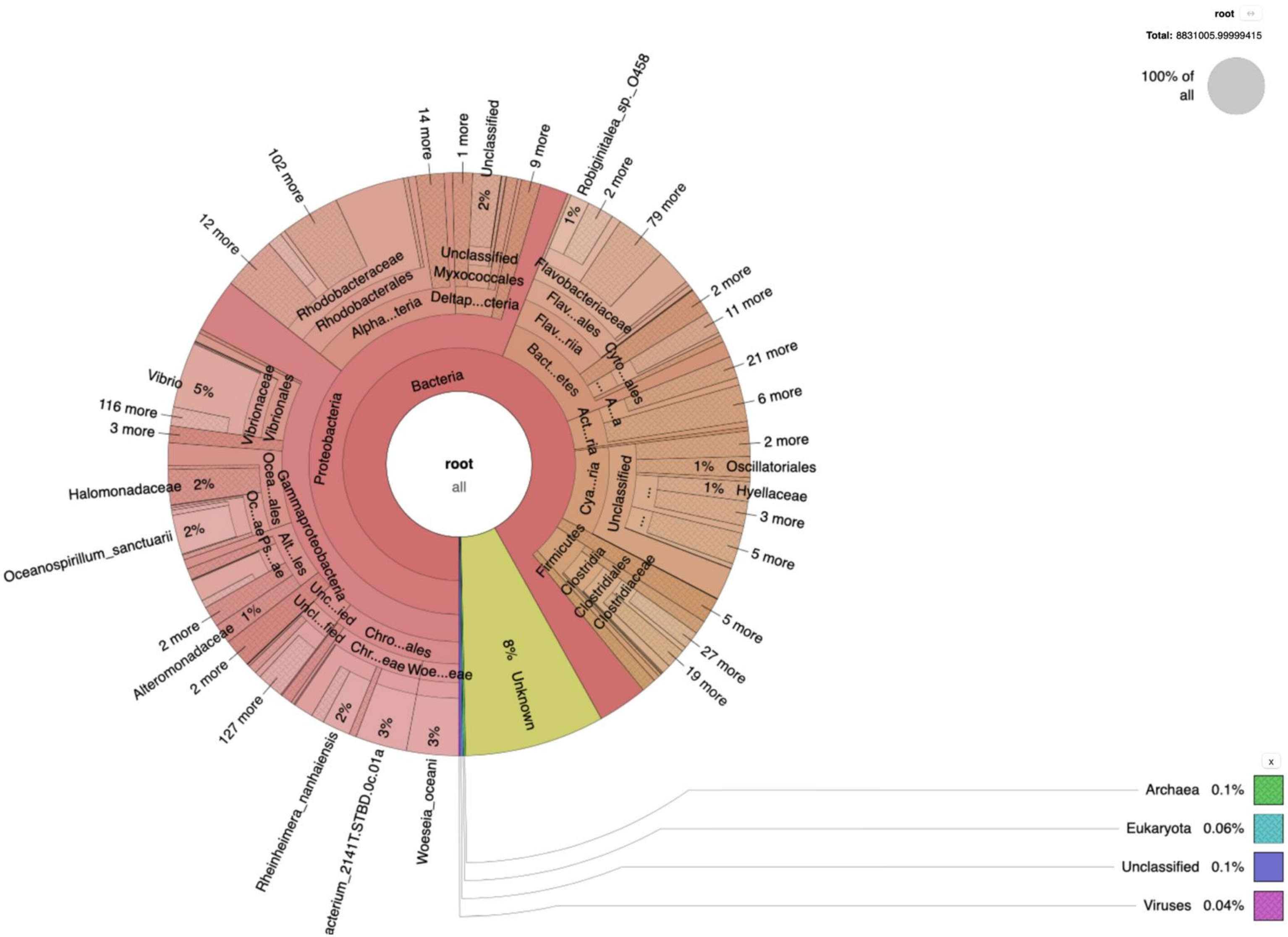


**Figure S2c. KRONA plot of sample S3**

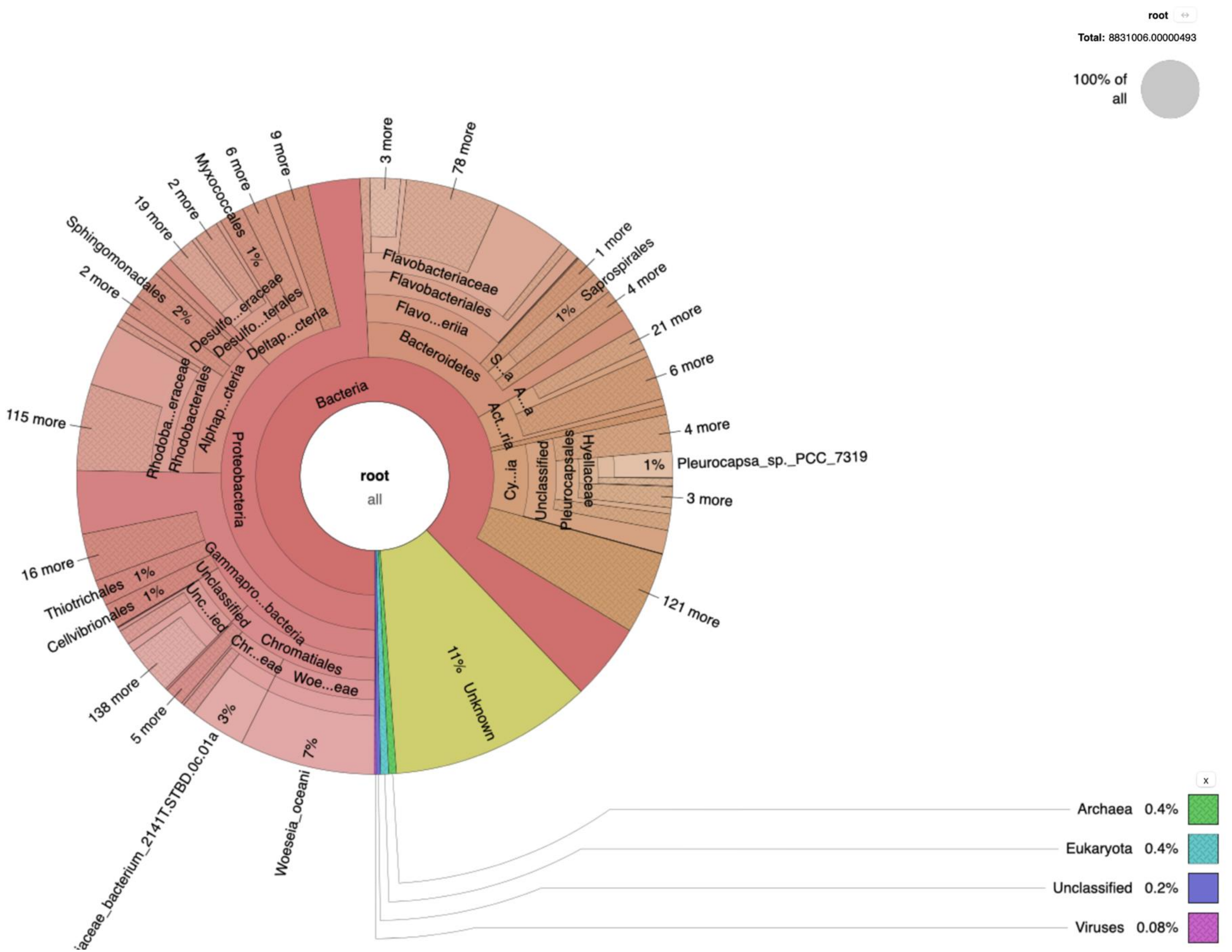


**Figure S2d. KRONA plot of sample S4**

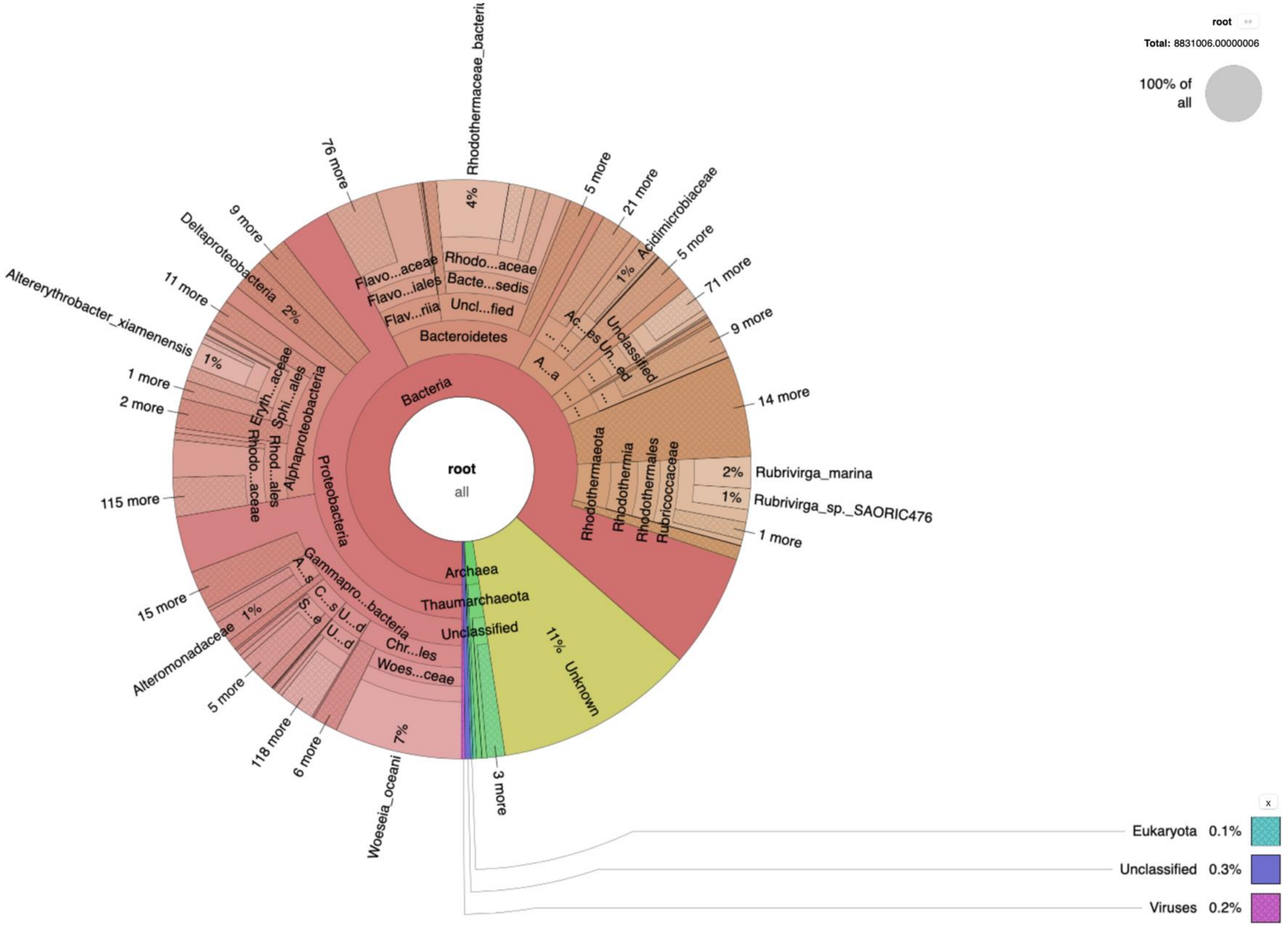


**Figure S2e. KRONA plot of sample S5**

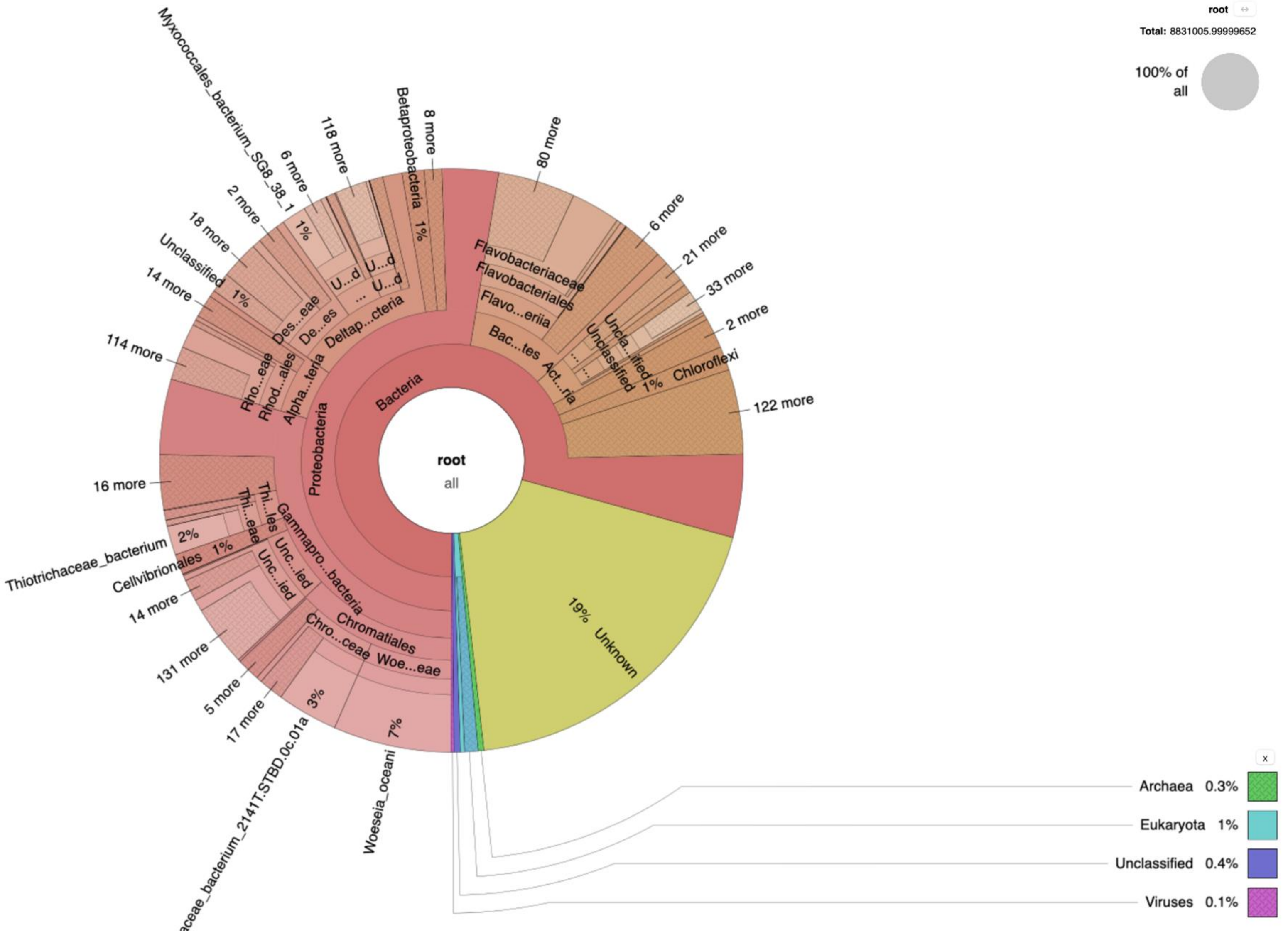


**Figure S2f. KRONA plot of sample S6**

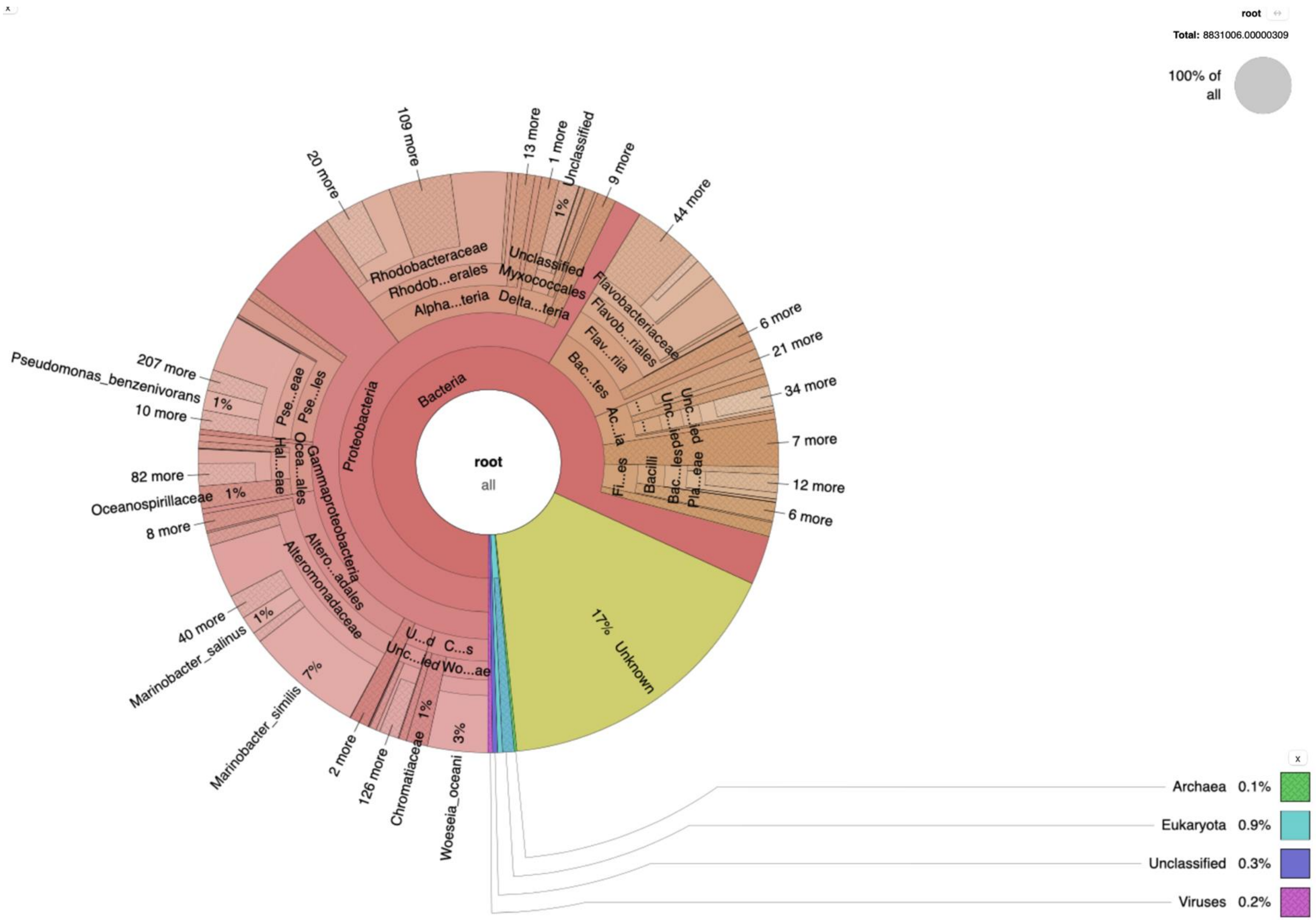


**Figure S2g. KRONA plot of sample S7**

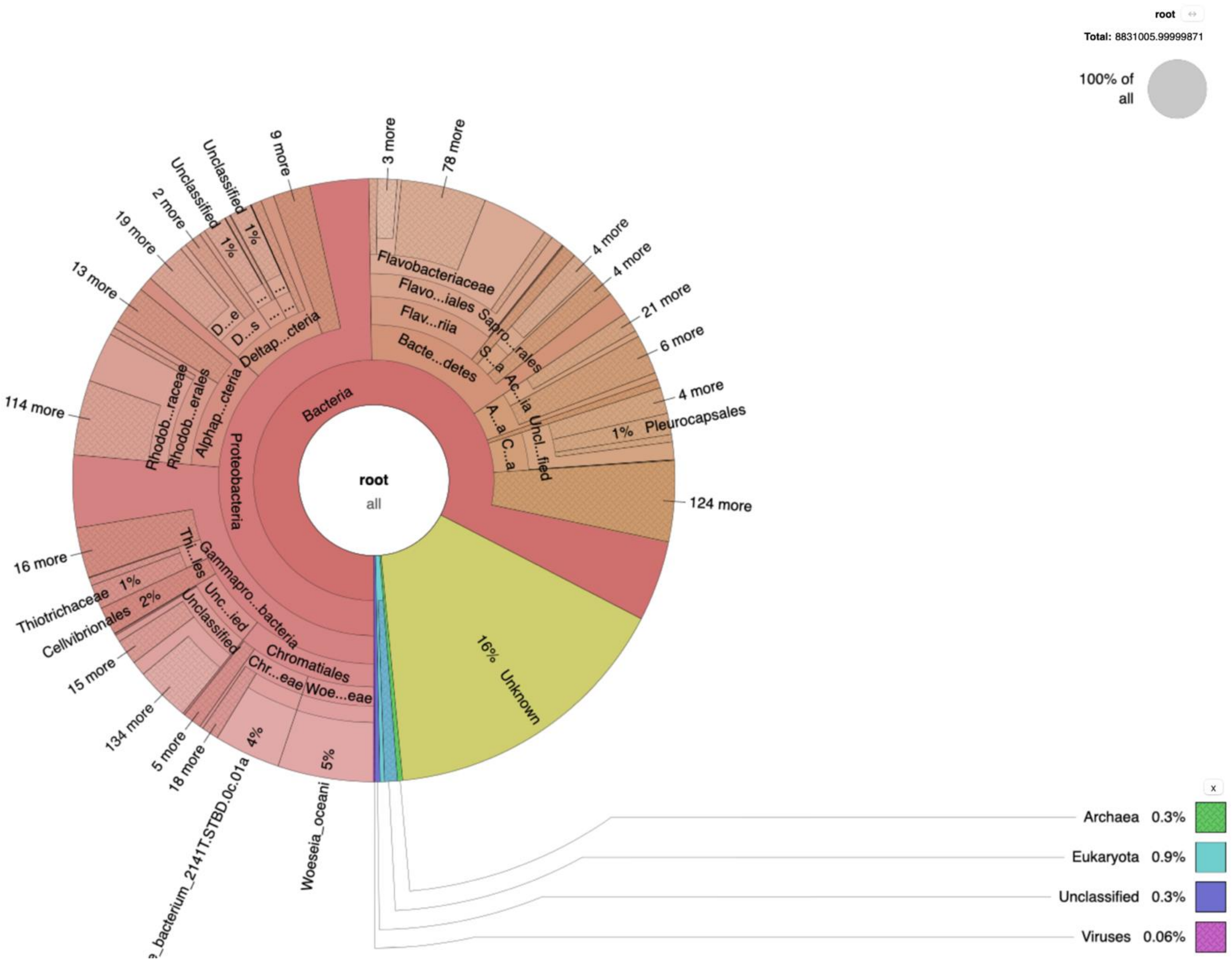


**Figure S2h. KRONA plot of sample S8**

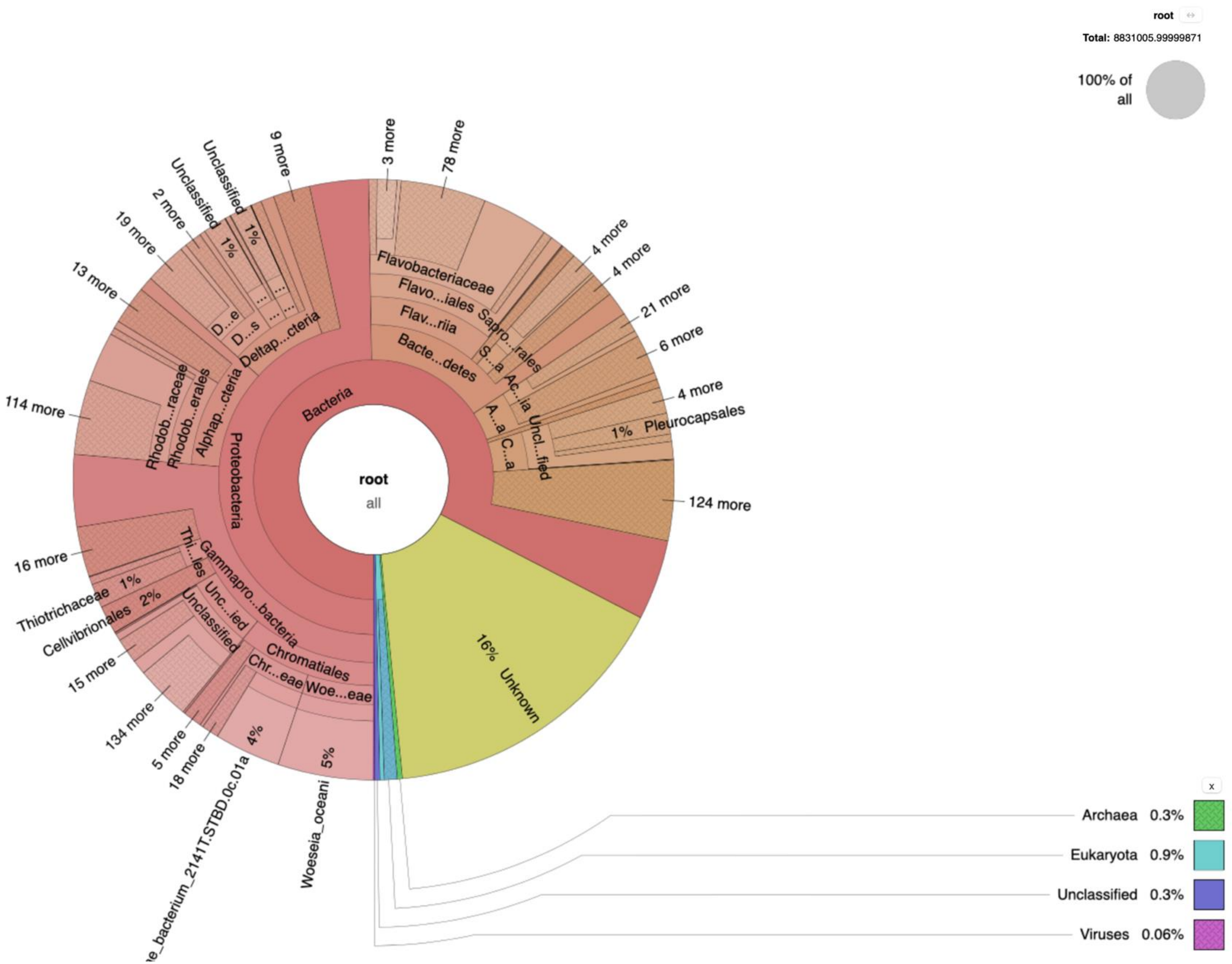


**Figure S2i. KRONA plot of sample S9**

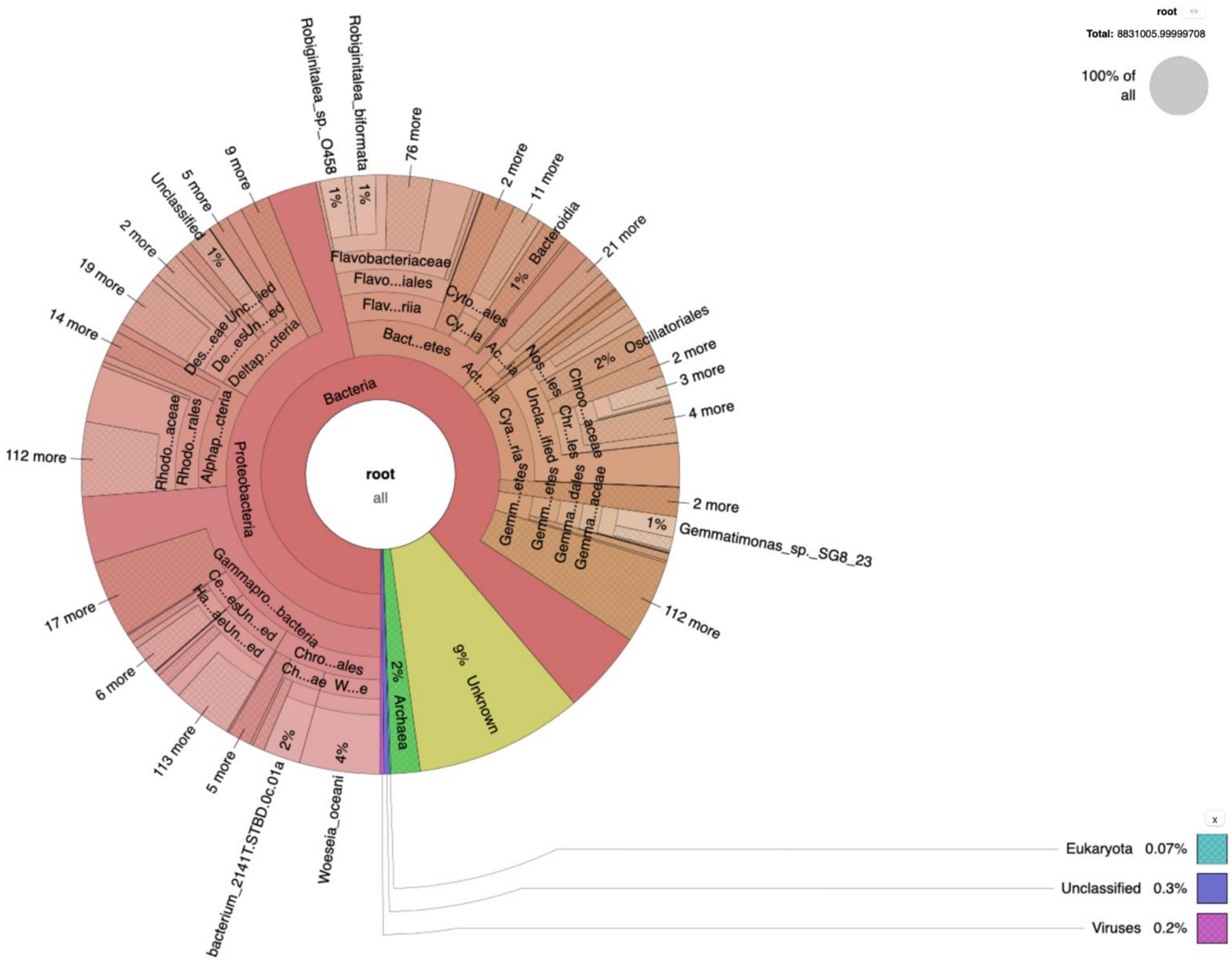


**Figure S2j. KRONA plot of sample S10**

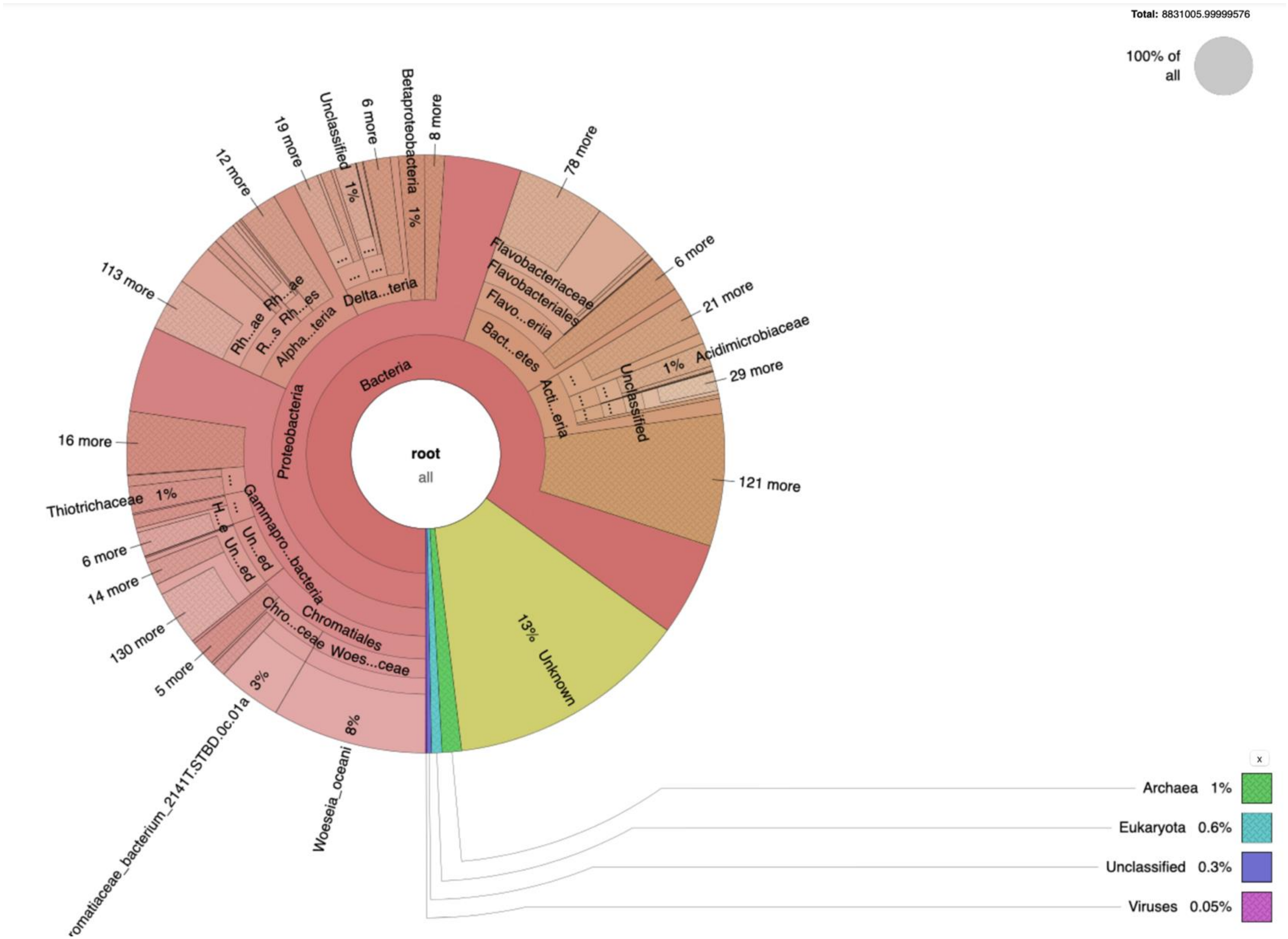


**Figure S2k. KRONA plot of sample S11**

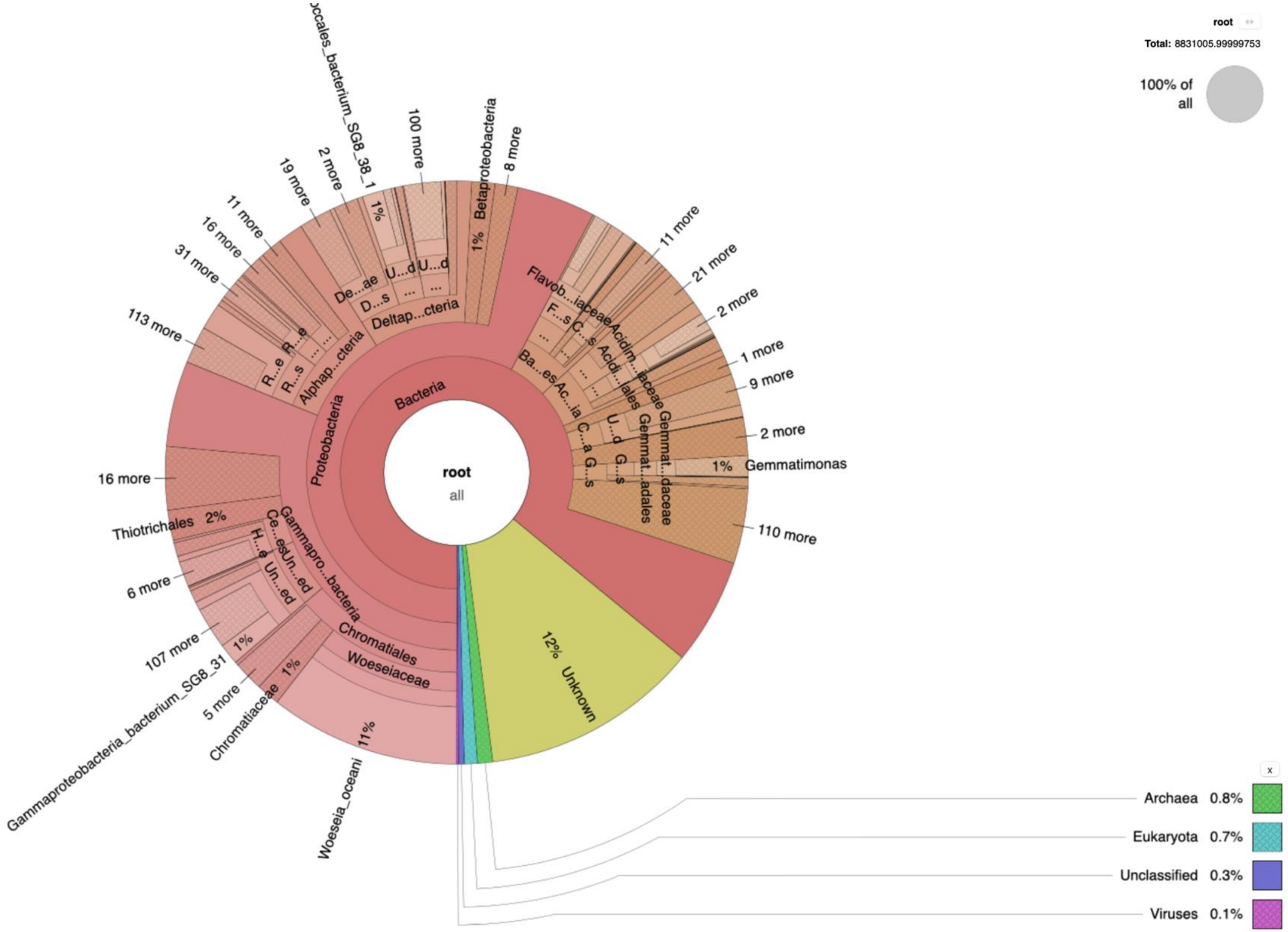


**Figure S2l. KRONA plot of sample S12**
